# Case report: Patient-derived organoids promoting personalized treatment in invasive urothelial carcinoma

**DOI:** 10.3389/fonc.2024.1424677

**Published:** 2024-11-01

**Authors:** Xun Liu, Xuebing Han, Shuqing Wei, Changwen Zhang

**Affiliations:** ^1^ Department of Urology, Tianjin Institute of Urology, The Second Hospital of Tianjin Medical University, Tianjin, China; ^2^ Department of Urology, Shanxi Province Cancer Hospital/Shanxi Hospital Affiliated to Cancer Hospital, Chinese Academy of Medical Sciences/Cancer Hospital Affiliated to Shanxi Medical University, Taiyuan, China; ^3^ Department of General Medicine, Shanxi Province Cancer Hospital/Shanxi Hospital Affiliated to Cancer Hospital, Chinese Academy of Medical Sciences/Cancer Hospital Affiliated to Shanxi Medical University, Taiyuan, China

**Keywords:** urothelial carcinoma, patient-derived organoids, neoadjuvant chemotherapy, treatment response, drug sensitivity testing

## Abstract

Tumor organoids, an *in-vitro* three-dimensional model, possess high potential for investigating tumor biology and treatment response and have been demonstrated more appropriate for drug assessment than two-dimensional cultures. Herein, we described two cases of invasive high-grade urothelial carcinoma who underwent radical cystectomy successfully following use of patient-derived organoids (PDOs) for drug screening to inform therapeutic decisions. In these two cases, the PDOs cultured by biopsy tissues were both sensitive to the combination of gemcitabine and cisplatin. After neoadjuvant chemotherapy (NAC) with gemcitabine and cisplatin, the patients responded well, and radical cystectomy was performed successfully. No recurrence or metastasis was observed within 6 months after surgery. This small case series suggests that the patient-derived urothelial carcinoma organoids contribute to optimizing NAC options to guide personalized treatment and improve the survival outcomes.

## 1 Introduction

Bladder cancer is a major health challenge worldwide, with a globally projected 573,278 new cases and 212,536 new deaths in 2020 (1). Urothelial carcinoma, known to have a wide range of molecular alterations and morphological characteristics, approximately accounts for 90% of all cases of bladder cancer in the USA and Europe (2). It is estimated that morphological variations can occur in about 15%-25% of invasive urothelial carcinoma in the form of “divergent differentiation”, accompanied by other epithelial lineages like trophoblastic, squamous, glandular, or small cell/high-grade neuroendocrine differentiation (3). Currently, radical cystectomy and pelvic lymph node dissection are the standard treatment modality for muscle-invasive bladder cancer. Although this treatment may be curative, a great number of patients will experience relapse.

Over the past decades, cisplatin-based neoadjuvant chemotherapy (NAC) has been demonstrated to improve the survival outcomes and has been proposed as the standard of care for muscle-invasive bladder cancer prior to radical cystectomy and pelvic lymph node dissection (4). Nevertheless, for nonresponsive patients they not only suffer substantial toxicity but also a delay in definitive local therapy. Tumor organoids, an *in-vitro* three-dimensional model, possess high potential for investigating tumor biology and treatment response, which are more appropriate for drug evaluation than two-dimensional cultures due to precise recapitulation of tissue architecture (5). Here, we described two cases of invasive high-grade urothelial carcinoma who underwent radical cystectomy successfully following use of patient-derived organoids (PDOs) for drug screening to inform therapeutic decisions.

## 2 Case presentation

### 2.1 Case 1

A 59-year-old man was admitted to the hospital due to intermittent painless gross hematuria for over 2 months. The patient experienced intermittent gross hematuria like meat-rinsing water without obvious predisposing causes 2 months before admission, accompanied by frequent urination, urgent urination, urodynia, incomplete emptying, and urine dripping. He had no fever, chill, nausea, vomiting, abdominal pain, diarrhea, and low back pain. Urinary ultrasound in the local hospital showed a space-occupying lesion in the bladder. On September 12, 2022, the patient was admitted to our hospital. He had no previous history of diabetes mellitus, coronary heart disease and cerebral infarction. Despite presence of hypertension for 20 years, he reported that his blood pressure was always under control.

**Figure 1:**
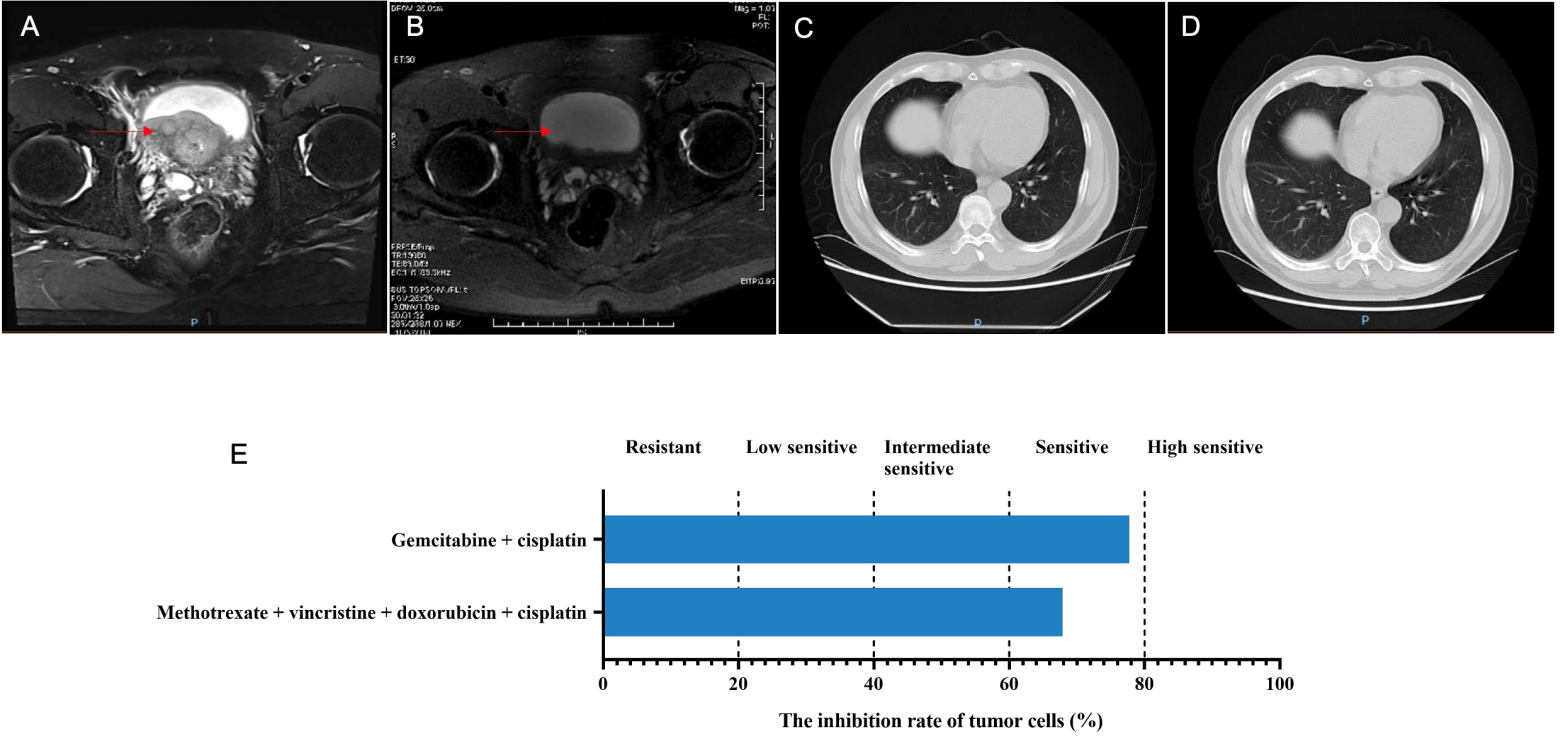
The disease changes of patient 1 based on MRI and CT scans, including initial diagnosis **(A)**, post-neoadjuvant chemotherapy **(B)**, 3 months after radical cystectomy **(C)**, 9 months after radical cystectomy **(D)**, as well as the PDO-based drug sensitivity testing results **(E)**. The red arrows point to the tumor.

**Figure 2:**
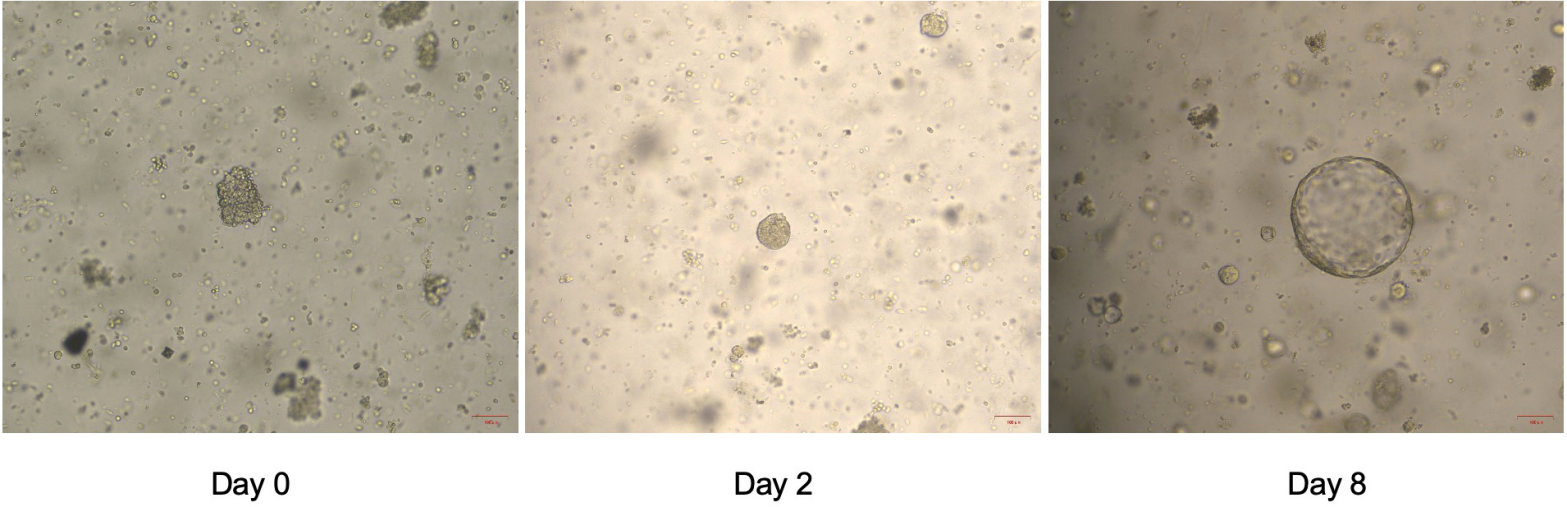
The status of organoid formation in patient 1 with invasive urothelial carcinoma on day 1, day 2 and day 8.

On September 13, 2022, the bladder magnetic resonance imaging (MRI) revealed multiple tumors invading the prostate in the urinary bladder (**Figure 1A**). The abdominal computerized tomography (CT) showed multiple space-occupying lesions in the bladder, various gallbladder stones, as well as duodenal and transverse colon diverticula. The chest CT indicated interstitial lesions in both lungs, with small nodules in the middle lobe of the right lung. Urinary cytology showed visible tumor cells. On September 14, 2022, transurethral resection of bladder tumors (TURBT) was performed under general anesthesia, during which multiple cauliflower-like masses with a broad base on the walls of the bladder and cauliflower-like masses in the urethra of the prostate were observed, thus multipoint electrosurgical biopsy was conducted. Through pathological examination, invasive high-grade urothelial carcinoma was diagnosed. Immunohistochemical results showed GATA3 (+), SMA (+), CD34 (vascular wall +), D2-40 (lymphatic wall +), and NKX3.1 (-). Postoperatively, gemcitabine (1 000 mg) was immediately used for intravesical therapy. Meanwhile, the patient’s tumor tissue was obtained for organoid culture. Briefly, the tumor tissue was minced and digested for 30 min after rinsing with precooled PBS, with the cells of 4.75×10^4^ achieved. Through centrifugation, cell pellets were collected. Subsequently, cells and Matrigel suspension were both seeded onto 6-well plates (2 mL per well) with pipettes after Matrigel was added, and placed in a 37°C incubator for 15 min. When droplets were solidified completely, the culture medium (Kingbio Medical [Chongqing] Co., Ltd., China) was added and cultured at an incubator (37°C, 5%CO_2_). The culture was terminated when organoids grew well and tended to stabilize, with the culture duration of 23 days. Through a microscope, it could be observed that the organoids manifested as solid, hollow or mixed spheroids. At this time, drug sensitivity testing was performed, which showed more sensitive to gemcitabine combined with cisplatin (**Figure 1E**). The status of organoid formation was presented in **Figure 2**.

On October 15, 2022, the NAC with gemcitabine (1 600 mg, intravenous drip, d1/8) and cisplatin (120 mg, intravenous drip, d2) was given, with 21 days as a cycle. After two cycles, the bladder MRI showed no obvious abnormally enhanced nodules or tumor shadows in the bladder, with dilation in the urethra and prostate (**Figure 1B**), which suggested complete response (CR). On November 26, 2022, the cystoscopy examination revealed no significant abnormalities in the urethral mucosae and bladder walls. Therefore, radical cystectomy with Bricker ileal conduit was performed on November 28, 2022. Pathological examination showed no residual tumors in the bladder tissue, but with a small number of histocytes and multinucleated giant cell reactions. Both ureteral ends were negative, and no metastases were found in the pelvic lymph nodes. One month after surgery, the patient continued to use gemcitabine and cisplatin for four cycles. The abdominal CT examinations showed no tumor recurrence or metastasis whether at 3 or 9 months after surgery (**Figures 1C, D**).

### 2.2 Case 2

A 47-year-old man was admitted to the hospital with the chief complaint of intermittent painless gross hematuria for over 2 months. The patient suffered from intermittent gross hematuria without obvious predisposing causes, sometimes with flaky blood blots and accompanied by frequent urination and urgent urination. He had no symptoms like pain and distension in the lower back, nausea, vomiting, fever, and chills. A space-occupying lesion in the bladder was observed by urinary ultrasound in the local hospital. On November 4, 2022, the patient came to our hospital for further treatment, without the history of diabetes mellitus, coronary heart disease, cerebral infarction, and surgery.

**Figure 3:**
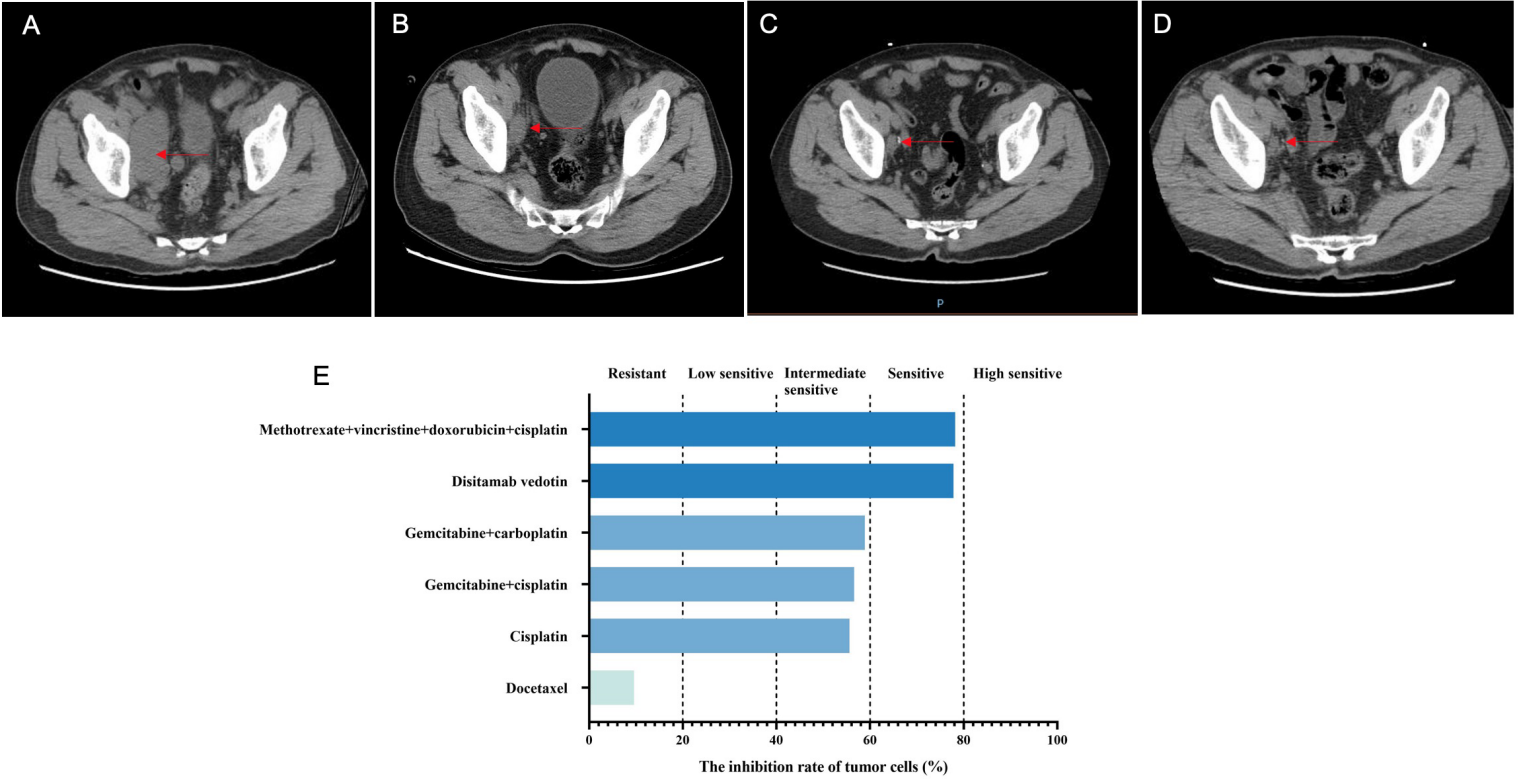
The disease changes of patient 2 using CT scans, including initial diagnosis **(A)**, post-neoadjuvant chemotherapy **(B)**, 3 months after radical cystectomy **(C)**, 6 months after radical cystectomy **(D)**, as well as the PDO-based drug sensitivity testing results **(E)**. The red arrows point to the tumor.

**Figure 4:**
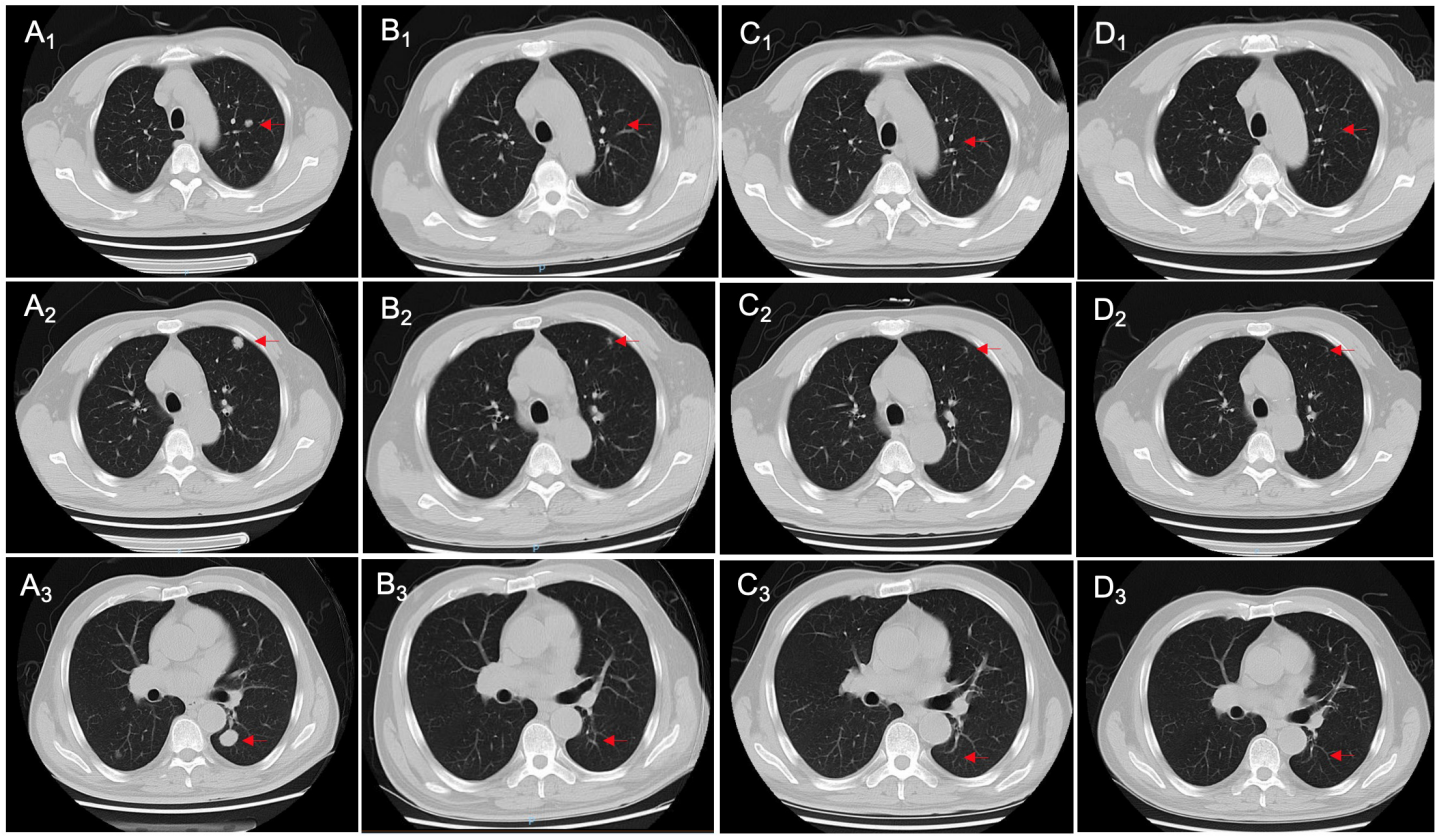
Changes in chest CT imaging of patient 2 during treatment, including initial diagnosis **(A_1_-A_3_)**, post-neoadjuvant chemotherapy **(B_1_-B_3_)**, 3 months after radical cystectomy **(C_1_-C_3_)**, and 6 months after radical cystectomy **(D_1_-D_3_)**. The red arrows point to the nodules.

After admission, the bladder MRI showed the tumors in the lower segment of the right ureter and bladder, accompanied by bilateral hydroureter and multiple lymphatic metastases. The abdominal CT indicated a space-occupying lesion in the bladder with bilateral hydroureter and hydronephrosis, as well as various nodules in the abdominal cavity, retroperitoneum, and pelvic cavity (**Figure 3A**). Additionally, multiple nodules were also observed in both lungs (**Figures 4A1–3**). Importantly, urinary cytology revealed visible tumor cells. On November 8, 2022, cystoscopy was performed, during which multiple cauliflower-like masses were found in the bladder. Based on the pathological examination, invasive high-grade urothelial carcinoma was suspected. Immunohistochemical results showed CK (+), CK20 (partial +), GATA3 (+), P53 (mutant), Ki-67 (approximately 95%+). After the right percutaneous nephrostomy performed on November 10, 2022 was used to eliminate hydronephrosis, the levels of creatinine and urea nitrogen were decreased from 93.3 to 80 μmol/L, and 6.5 to 6 mmol/L, respectively. Four days later, their levels (creatinine, 59.3 μmol/L; urea nitrogen, 5.9 mmol/L) were further reduced. As described in case 1, the patient’s tumor tissue was collected for organoid establishment in Kingbio Medical (Chongqing) Co., Ltd., China. After 30-min digestion, the cells of 7.71×10^4^ was obtained. For this patient, the organoid culture duration was 22 days. After culture termination, drug sensitivity testing was performed. The results showed that gemcitabine combined with cisplatin might be the potential candidates for the patient (**Figure 3E**).

On December 13, 2022, the NAC with gemcitabine (1 600 mg, intravenous drip, d1/8) and cisplatin (120 mg, intravenous drip, d2) was administrated, with 21 days as a cycle. After two cycles, the abdominal CT showed alleviations in thickening of the bladder wall, dilation of bilateral ureters and pyelocaliectasis, as well as a decrease in the number and size of multiple nodular shadows and slightly low-density shadows in the pelvic cavity (**Figure 3B**). Moreover, some nodular shadows in both lungs also diminished (**Figures 4B1–3**). Gemcitabine combined with cisplatin continued to be used for one cycle. On February 14, 2023, the bladder MRI revealed no obvious masses in the right lower ureter and bladder, without dilated bilateral ureters and significantly reduced pelvic lymph nodes. Therefore, radical cystectomy and cutaneous ureterostomy were performed. Postoperatively, there was no cancer cell invasion found in bilateral urethral broken ends and the broken ends of bilateral seminal vesicles and seminiferous ducts, without metastases in the right pelvic lymph nodes. Subsequently, gemcitabine combined with cisplatin was used as adjuvant chemotherapy. At 3 and 6 months after surgery, the ductal shadows leading out of the body were observed in both ureters, with mild pyelocaliectasis (**Figures 3C, D**). No significant increase was found in the nodules of both lungs (**Figures 4C1-3, D1-3**).

## 3 Discussion

Bladder cancer is one of the most prevalent and highly heterogeneous malignancies, with relatively poor outcomes. Although chemotherapy is effective in a subset of patients, it results in severe toxicity. At present, the treatment decision-making is mainly based on evaluating a single fragment of surgically obtained tumor tissue. For bladder cancer novel preclinical models are essential for making patient-specific treatment strategies targeted to tumor features. The patient-derived tumor organoid, a preclinical model with improved biomimicity, has emerged as a powerful tool to study the tumor evolution and treatment response. A systematic review has demonstrated a high concordance between bladder cancer organoids and their primary tumor tissues at histological, genetic, and phenotypic levels (6). In this case series, we first described two cases of invasive high-grade urothelial carcinoma who successfully underwent radical cystectomy after use of the NAC with gemcitabine and cisplatin which were shown sensitive in the PDOs for drug screening, highlighting enormous potential of the PDOs in optimizing the NAC options in invasive urothelial carcinoma.

Bladder cancer is commonly diagnosed by biopsies acquired through a cystoscope entering the bladder, in which muscle-invasive or metastatic bladder cancer approximately accounts for 27%. Despite refinement of surgical techniques and improvement of anatomical knowledge, the prognosis of patients with muscle-invasive bladder cancer including metastatic diseases remains to be poor, with the 5-year survival rate of about 35% (7). There is strong evidence that multimodality treatment composed of cisplatin-based NAC followed by radical cystectomy or radiotherapy contributes to improving the outcome of high-risk patients with muscle-invasive bladder cancer, although an increased rate of 6.5% at most in overall survival through a 5-year follow-up visit (8, 9). However, one of the defects of NAC is the uncertainty to chemosensitivity. In muscle-invasive bladder cancer, the pathological complete response rate is only about 25%, suggesting that up to 75% of patients would not derive benefits from this treatment (8). Therefore, it is indispensable to assess the tumor biology and chemosensitivity of patients with bladder cancer at individual levels to guide the personalized treatment of bladder cancer.

In recent years, organoids as the 3D miniature structures have emerged, which provides novel insights for studying tumor evolution and assessing treatment response. Unlike cell lines, the PDOs can highly recapitulate the histological architecture, gene expression and genomic alterations of the original tumors, which not only contributes to screening new anti-cancer drugs, but also reflects the patient’s clinical response based on drug sensitivity testing (10). Although patient-derived tumor xenografts have been demonstrated highly effective in predicting the efficacy of conventional and new anti-cancer drugs (11), they have disadvantages of low transplantation success rates, long culture time and high cost (12). Currently, the organoids from multiple tissues, such as bladder, stomach, breast and more, have been cultured successfully (13). In a previous study, the PDOs from bladder cancer have been demonstrated to retain the broad mutational, molecular, and histopathologic profiles of the parental tumors, including non-muscle invasive and muscle invasive bladder cancers (14). They could be not only retained for prolong periods of time, but also closely resemble the tumor histology. Importantly, bladder cancer organoids can also preserve the tumor heterogeneity and mutational burden, appropriate for drug screening (15). For the comparison of standard-of-care drugs with other chemotherapy drugs, use of bladder cancer organoid-based drug screening was demonstrated to be feasible. In the latest PDO-related expert consensus, it has been proposed that PDOs can be utilized to predict clinical responses of patients with multiple cancer types including urothelial cancer (16, 17). In our case series, the PDOs from urothelial carcinoma both showed sensitive to gemcitabine combined with cisplatin. After treatment with gemcitabine and cisplatin, these two cases both achieved good response and underwent radical cystectomy, without recurrence or metastasis after surgery. These findings suggest that bladder cancer organoids can be used to optimize the NAC options, thus promoting the improvement of the patient’s prognosis. Meanwhile, the PDOs can help to exclude ineffective drugs or treatment options for patients through high-throughput screening while quickly identifying highly sensitive drugs, thus providing more reliable data for customized treatments. Importantly, PDO-based drug sensitivity testing has been demonstrated to be implemented throughout the entirety of cancer treatment, including neoadjuvant and/or palliative chemotherapy, ineffective first-line treatment, etc. (16). However, our study belonged to case-series study, and only two cases were enrolled, which may weaken the strength of our evidence to a certain extent. Additionally, for the assessment of the long-term prognosis, the follow-up duration of these two patients was relatively short. In the future, we will conduct the large-scale studies with long follow-up duration to further validate our findings.

In conclusion, this small case series first demonstrates the enormous potential of the patient-derived urothelial carcinoma organoid in guiding the selection of NAC options, further unveiling that bladder cancer organoids used for drug screening can be a powerful tool for optimizing NAC options to facilitate the personalized treatment of patients with bladder cancer.

## Data Availability

The original contributions presented in the study are included in the article/supplementary material, further inquiries can be directed to the corresponding author/s.
